# Efficacy of switching treatment to faricimab in recalcitrant neovascular age related macular degeneration – 6 month results after completion of the loading phase

**DOI:** 10.1007/s00417-025-06903-9

**Published:** 2025-07-31

**Authors:** Franziska Eckardt, Michael Hafner, Anna Lorger, Caspar Liesenhoff, Johannes Schiefelbein, Tina Rieke Herold, Nikolaus Luft, Julian Elias Klaas, Benedikt Schworm, Siegfried Georg Priglinger, Jakob Siedlecki

**Affiliations:** Department of Ophthalmology, LMU University Hospital, LMU Munich, Munich, Germany

**Keywords:** Age related macular degeneration, Anti-VEGF, Retina, Biomarker

## Abstract

**Purpose:**

To report the efficacy and durability of switching treatment to faricimab in recalcitrant neovascular age-related macular degeneration (nAMD) at six months after completion of the loading phase (nine months after switching).

**Methods:**

Recalcitrant nAMD was defined as persistent fluid despite monthly injections (q4w) or inability to extend treatment intervals beyond six weeks (q6w). The study included patients on a treat & extend regimen for six months after three monthly injections. Primary outcomes were changes in central subfield thickness (CST), subfoveal choroidal thickness (SFCT), visual acuity, and injection interval.

**Results:**

Nine month-data was available for 56 eyes initially switched to faricimab. At nine months, 51 eyes (91.1%) of 49 patients were maintained on faricimab, while five eyes (8.9%) had been switched back to their older agent. At nine months after switching, median CST was significantly reduced as compared to baseline at switching (332,00 (Q1:295,00; Q3:394,00) to 303,00 (Q1:269,00; Q3:366,00) µm; *p* < 0.001). Median SFCT also decreased from 158,00 (Q1:116,00; Q3:219,00) µm to 127,00 (Q1:95,00; Q3:196,00) µm (*p* < 0.001). The average injection interval was significantly extended from 37.0 ± 9.5 days prior to switching to 56.1 ± 30.4 days at nine months (*p* = 0.002). Visual acuity was maintained (0.30 (Q1:0.10 Q3:0.50) vs. 0.30 (Q1:0.10 Q3:0.50) logMAR; *p* = 0.07).

**Conclusion:**

In recalcitrant nAMD, faricimab can improve CST and SFCT while maintaining visual acuity. Furthermore, greater durability could be achieved with faricimab at nine months as compared to ranibizumab or aflibercept. Further prospective randomized trials are warranted.

## Key messages

*****What is known***:**

- Phase three studys showd promising results of farcimab treatment in treatment naiv eyes.
- Preclinical studies revealed positive results with regard to dual pathway inhibition.
- Analyses of the loading phase indicated generally good responses after switching to faricimab.

*****What is new***:**

- Switching to farcimab also achieves positive effects in pre-treated eyes.
- Positive effects on the biomarkers of AMD are also observed 9 months after switching, with a significant extension of the treatment interval. The visual acuity remains stable.

## Introduction

Age-related macular degeneration (AMD) is one of the leading causes of blindness in developed countries, with especially aggressive disease dynamics in its neovascular phenotype (nAMD) [1–3]. NAMD activity is usually defined by increasing central retinal thickness (CST), new intraretinal macular fluid (IRF), new subretinal fluid (SRF), new sub-retinal pigment epithelial fluid (sub-RPEF) or progressive epithelial detachment (PED), all of which derive from exudation from pathologic macular neovascularizaton [4–6].

Vascular endothelial growth factor (VEGF) is crucial in the pathogenesis of nAMD. The advent of intravitreal anti-VEGF agents marked a significant breakthrough in nAMD therapy, as they suppress endothelial cell proliferation, vascular permeability, and all the fibrovascular complications associated by the outgrowth of MNV [4, 7]. Within the therapeutic armamentarium of anti-VEGF inhibitors, ranibizumab (Lucentis^®^, Novartis, Basel, Switzerland), aflibercept (Eylea^®^, Bayer, Leverkusen, Germany), brolucizumab (Beovu^®^, Novartis) and bevacizumab (off-label use, Avastin^®^, Roche, Grenzach-Wyhlen, Germany) mainly focus on a single-target strategy [8].

On the contrary, faricimab (Vabysmo^®^, Roche) represents a novel bispecific anti-VEGF/Angiopoietin 2 (Ang-2) inhibitor which was approved by the FDA and EMA in 2022 for the treatment of nAMD. By promoting vascular destabilization and enhancing VEGF signaling mediated by the additional Tie-2 pathway, Ang-2 strongly contributes to the pathological neovascular processes observed in nAMD [9]. The potential of an Ang-2 inhibitor is therefore to decrease vascular remodeling and stabilize the vasculature. By targeting both VEGF and Ang-2, Faricimab offers a dual mechanism of action, potentially providing more effective inhibition of the neovascular processes in nAMD compared to drugs that target VEGF alone [7, 10]. Several studies have demonstrated the safety and efficacy of faricimab [11–14]. Based on the phase 3 studies LUCERNE and TENAYA, faricimab may enable more efficient disease management by extending the injection interval to up to 16 weeks after a loading phase of four monthly injections [11].

For patients suffering from an inadequate treatment response, i.e. persistent macular fluid despite monthly dosing or the inability to extend treatment intervals beyond 4 to 6 weeks, this dual mode of action might provide benefits [5, 15]. Given the, however, relatively short period of time since approval in 2022, there are currently few clinical trials investigating the real world long-term efficacy and durability of faricimab in switch patients. Therefore, this retrospective real-world study was designed to investigate the efficacy and durability of switching treatment to faricimab in recalcitrant nAMD at six months after completion of the loading phase. We aimed to assess whether treatment with faricimab in recalcitrant nAMD could provide better anatomical control and greater durability reflected by longer injection intervals.

## Methods

### Participants

For this retrospective study, we screened the Smart Eye Database (SmEyeDat) of the Department of Ophthalmology at LMU Munich for patients undergoing faricimab treatment for neovascular AMD between October 2022 and April 2024. Inclusion criteria comprised: (i) switch to faricimab after prior intravitreal therapy for nAMD due to a suboptimal response to ranibizumab/aflibercept treatment, defined by the persistence of intraretinal/subretinal fluid despite monthly anti-VEGF therapy or the inability to extend injection intervals beyond six weeks (fluid relapse after seven weeks); (ii) completion of a faricimab loading phase with three monthly injections after the switch with treat & extend treatment thereafter; (iii) absence of confounding factors such as intraocular infection or uveitis. Retreatment decisions were based primarily on OCT parameters, particularly the presence of intraretinal or subretinal fluid. Functional criteria such as changes in best-corrected visual acuity or patient-reported symptoms were considered supportive but not decisive in treatment interval adjustment. The study received approval from the ethics committee of the Medical Faculty of LMU Munich (study number 24–0638) and adhered to the principles of the Declaration of Helsinki. All patients provided written informed consent for intravitreal injection. Epidemiological data including age, gender, date of initial nAMD diagnosis, extent of prior therapy with intravitreal injections, and date of transition to faricimab were collected for each patient. Due to its retrospective design, the study may be subject to inherent limitations such as selection bias, lack of randomization, and variability in clinical documentation.

### Multimodal imaging

Multimodality imaging was performed as necessary and included SD-OCT and near-infrared (NIR) scans with Heidelberg Engineering’s Spectralis HRA + OCT system at each visit. At initial diagnosis, fluorescein angiography was performed prior to initiation of treatment. OCT data were collected from the time of treatment initiation at nAMD (T1), the last three visits with intravitreal injections before switching to faricimab (T2-T4), after the first three intravitreal faricimab injections of the loading phase (T5-T7) and the time approximately 6 months after upload completion (T8). Automated CST measurements were performed using Heidelberg Eye Explorer (version 1.10.12.0) after manually correcting the segmentation if necessary. The subfoveal choroidal thickness (SFCT) was measured underneath the fovea in 1:1 μm imaging mode from the outer part of the retinal pigment epithelium to the sclerochoroidal interface. The EDI mode (enhanced depth imaging) was also switched on if the chorioscleral border could not be visualized due to a thick choroid.

### Anti-VEGF treatment

Previous injection therapy involved aflibercept (subgroup A) or ranibizumab (subgroup R). All patients received a faricimab load of three injections 30 days ± 7 days apart, after which the interval was extended according to the treat & extend regimen. If no disease activity was seen, intervals were extended by two weeks; if MNV activity was diagnosed on OCT, the interval was shortened by two weeks. MNV activity was defined as new or increasing macular fluid (IRF and SRF) or PED, or increasing CST.

### Visual acuity measurements

Visual acuity (VA) was recorded with in decimal VA and then converted to logarithm of minimum angular resolution (logMAR) units for analysis.

### Data analysis and statistics

Microsoft Excel version 16.72 for Mac was used for data management. All statistical analysis was performed in IBM SPSS^®^ Statistics 28 (IBM Deutschland GmbH). The significance level was set at *p* < 0.05. No normal distribution was found using the Shapiro-Wilk test. Further, the Friedman test was used to analyze the data during the treatment period. A post-hoc analysis was then performed for the pairwise comparison of the values. The Cohen’s effect size of the Friedman test was calculated using r = (z/√n), where *r* < 0.3 indicates a weak effect, 0.3–0.5 a medium effect and > 0.5 a strong effect.

## Results

### Baseline demographics

In total, 61 eyes of 56 patients with recalcitrant nAMD were switched to faricimab during the investigated timespan. Of those, five eyes (8.2%) were excluded from the study due to loss to follow-up at nine months. Of the remaining 56 remaining eyes, five eyes (8.9%) had been switched back to their older agent at the treating physician’s discretion at nine months. In all five cases, the decision to switch back to the previous agent was due to a lack of anatomical response and thus the inability to extend the treatment interval. The decision was made in agreement with the patient. Thus, 51 eyes (91.1%) of 49 patients were maintained on faricimab at nine months and were included in this study.

Baseline demographics are summarized in Table 1. Average age at nAMD diagnosis was 74.5 ± 6.8 years, there were 28 women and 23 men. On average, patients received 31.9 ± 24.6 anti-VEGF injections prior to switching, consisting of 13.0 ± 13.1 ranibizumab and 19.1 ± 19.4 aflibercept injections on average. The 6-month analysis was performed 189.4 ± 16.6 days after the completion of the loading phase. There were no severe ocular complications recorded during this study period (no cases of intraocular inflammation, retinal detachment, severe spikes in IOP, intraocular hemorrhages or tears of the retinal pigment epithelium).

**Table 1:** Baseline demographics, including age, gender, MNV type and prior intravitreal injection therapy

Number of patients	49
Number of eyes	51
right	25
left	26
Mean Age (years)	74.5 ± 6.8
Gender	
male	23
female	38
MNV type	
1	21 (41.2%)
2	20 (39.2%)
3	10 (19,6%)
OCT findings at the day of switch	
CST (µm)	391.15 ± 148.89
SFCT (µm)	172.33 ± 77.40
IRF (n)	27 (52.9%)
SRF (n)	39 (76.5%)
PED (n)	31 (80.4%)
Mean prior anti-VEGF injections	
Total (n)	31.88 ± 24.55
Total RBZ	13.04 ± 13.14
Total AFL	19.10 ± 19.43
Mean injections/year	8.99 ± 2.56
Last injection	
Ranibizumab	13 (25.5%)
Aflibercept	38 (74.5%)
Injection interval before switch (days) Injection interval after switch (days)	37.0 ± 9.6 56.1 ± 30.4

*MNV* macular neovascularization, *OCT* optical coherence tomography, *SRF* subretinal fluid, *IRF* intraretinal fluid, *PED* pigment epithelium detachment, *CST* central subfield thickness, *SFCT* subfoveal choroidal thikness, *RBZ* Ranibizumab, *AFL* Aflibercept

## Fluid, CMT and SFCT dynamics

**Fig. 1:**
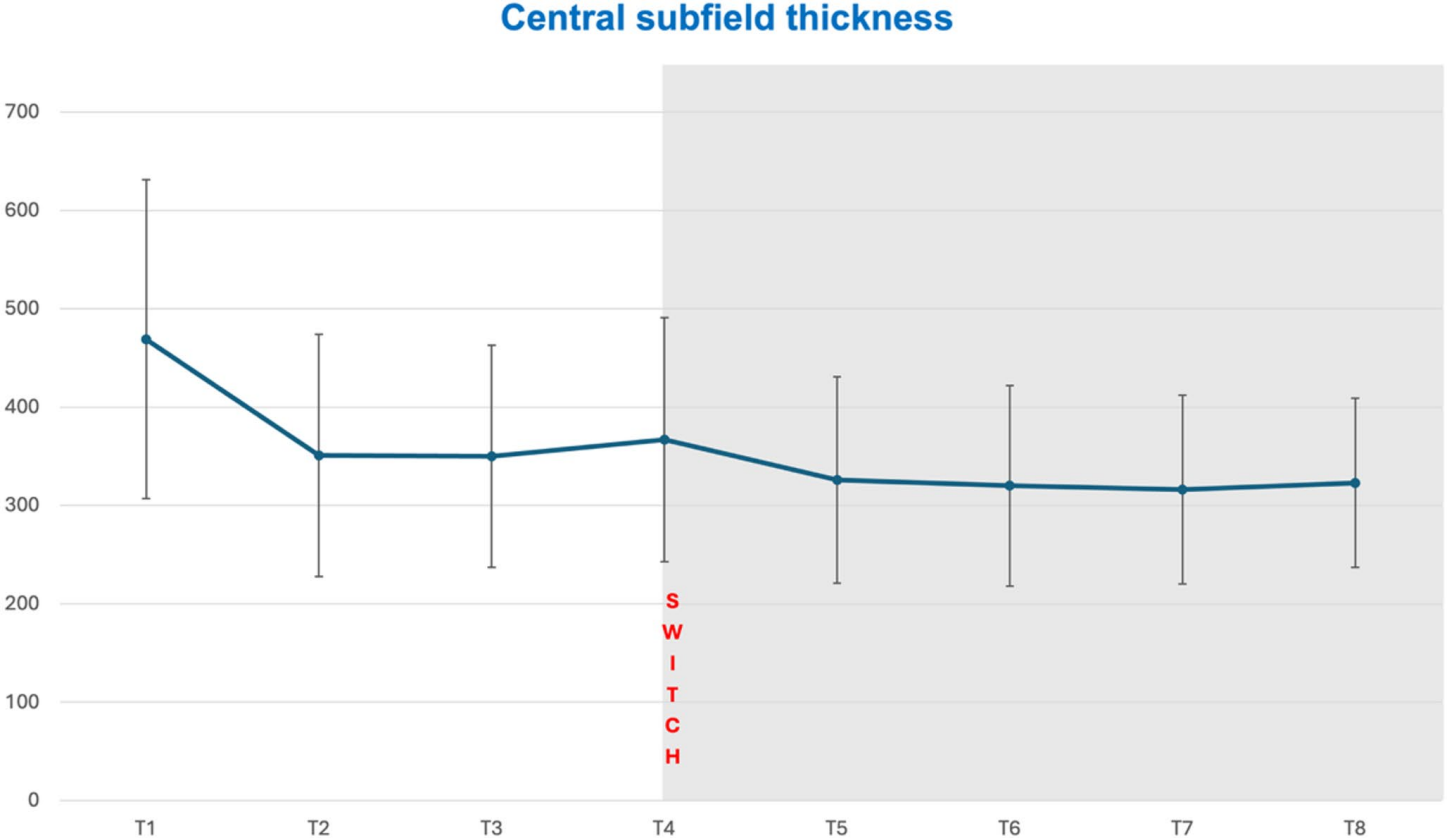
Central subfield thickness (CST) progress before (T2-T4) and after (T5-T8) switch to faricimab (T4)

**Fig. 2:**
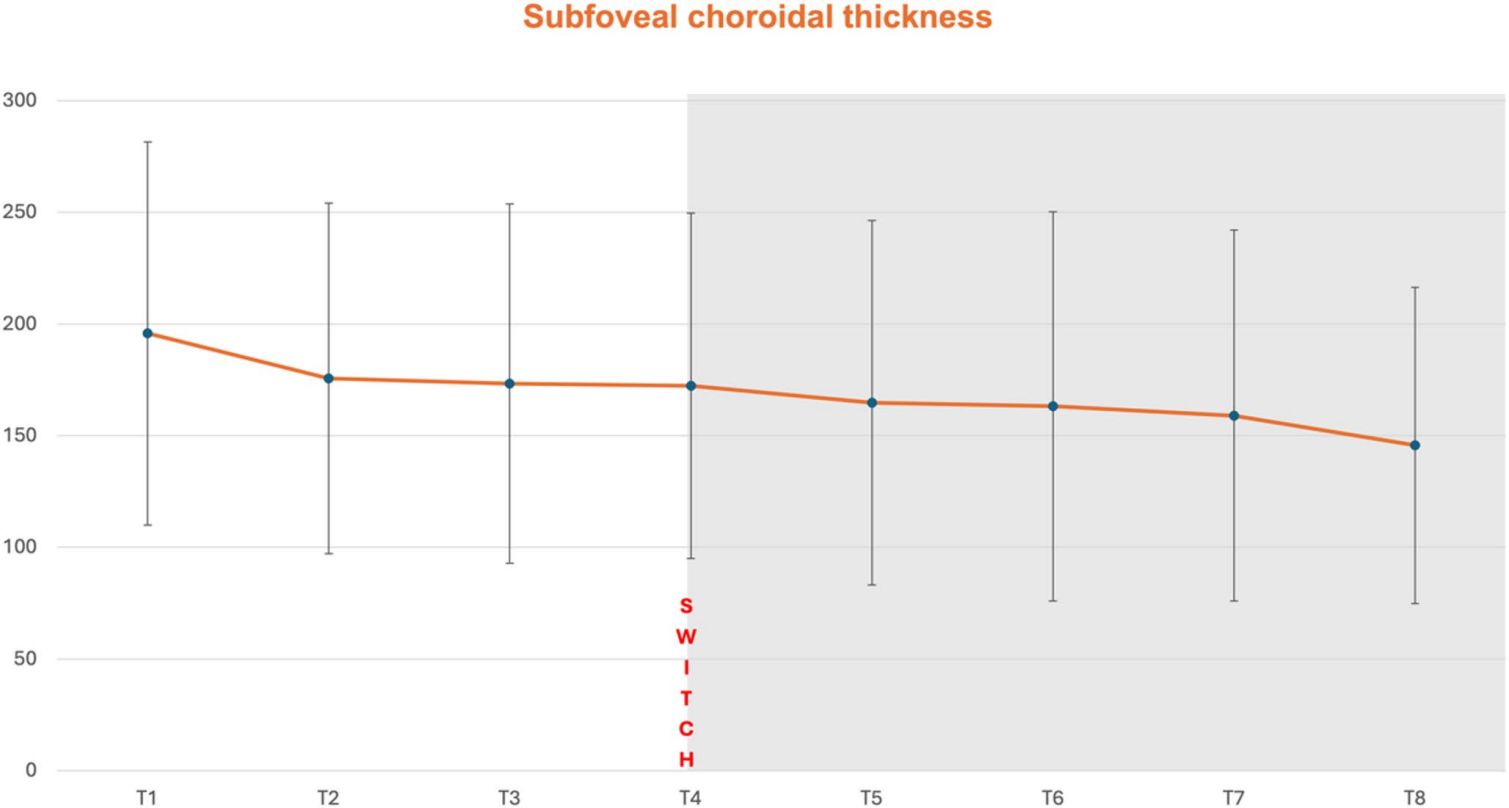
Subfoveal choroidal thickness (SFCT) progress before (T2-T4) and after (T5-T8) switch to faricimab (T4)

At initial diagnosis, MNV activity was detected in all eyes with IRF/SRF or PED. CST measurements at each visit are shown in Tables 2 and 3. Paired comparison of Friedman Test showed a significant difference between measurements during T1-T8 (*p* < 0.01). Under prior treatment between initial diagnosis T1 and T4 (*p* = 0.271) and between T2 and T4 (*p* > 0.99) no significant CST reduction was achieved. During the faricimab loading phase (T4-T7) CST decreased significantly (*p* < 0.001). Six months after the loading phase at T8, CST did not improve significantly any further compared to T7 (*p* = 0.095). However, compared to T4, CST was still significantly reduced (*p* < 0.001). At month nine, 12 patients showed an increase in CST; in 9 cases this was minor (< 50 μm), while in 3 patients, CST increased by more than 50 μm and had persistend IRF. Notably, these three patients had previously received over 20 intravitreal injections of other agents and were diagnosed with type 3 MNV. Dynamics of CST during the study period are shown in Figs. 1 and 2.

**Table 2:** Central subfield thickness (CST) measurements during study period (T1-T8)

Time points	CST (µm) all eyes (*n* = 51)	CST (µm) ranibizumab subgroup (*n*=13)	CST (µm) aflibercepts subgroup (*n*= 38)
T1	468.55 ± 161.91 428,00 (Q1:342,00; Q3:563,00)	450.38 ± 162.07 401,00 (Q1:337,00; Q3:586,50)	474.76 ± 163.55 448,00 (Q1:346,75; Q3:566,00)
T2	351.49 ± 123.06 314,00 (Q1:282,00; Q3:377,00)	367.77 ± 139.19 308,00 (Q1:274,00; Q3:468,00)	345.92 ± 118.56 322,00 (Q1:290,75; Q3:370,75)
T3	350.02 ± 113.46 327,00 (Q1:290,00, Q3:370,00)	379.15 ± 139.38 330,00 (Q1:282,50; Q3:445,00)	340.05 ± 103.42 320,50 (Q1:290,00; Q3:357,00)
T4	466.82 ± 123.63 332,00 (Q1:295,00; Q3:394,00)	391.15 ± 148.89 321,00 (Q1:285,00; Q3:564,00)	358.50 ± 114.83 334,50 (Q1:291,75,; Q3:392,50)
T5	325.71 ± 104.90 297,00 (Q1:266,00; Q3:350,00)	344.46 ± 136.73 287,00 (Q1:255,00; Q3:434,50)	319.29 ± 92.95 298,50 (Q1:272,00; Q3:329,50)
T6	319.69 ± 102.10 294,00 (Q1:266,00; Q3:341,00)	335.15 ± 128.05 271,00 (Q1:260,00; Q3:401,50)	314.39 ± 93.04 294,00 (Q1:269,50; Q3:336,50)
T7	315.90± 96.00 297,00 (Q1:266,00; Q3:335,00)	334.38 ± 121.55 299,00 (Q1:266,50,; Q3:342,00)	309.58 ± 77.74 294,50 (Q1:264,00; Q3:335,25)
T8	322.65 ± 85.78 303,00 (Q1:269,00; Q3:366,00)	344.69 ± 106.36 349,00 (Q1:262,50; Q3:386,50)	315.11 ± 77.74 303,00 (Q1:273,25; Q3:348,25)

top row: mean; bottom row: median (Q1 = 1^st^ Quartile; Q3 = 3^rd^ Quartile)

**Table 3:** Central subfield thickness in pairwise comparison

Time points	Interpretation CST	Pairwise comparison Asymptotic *p*- value	Effect power
T4 – T5	prior switch – after first faricimab injection	<0.001**	**0.4**
T4 – T7	prior switch – after third faricimab injection	<0.001**	**0.5**
T4 – T8	prior switch – third faricimab injection vs. 10-month FU	<0.001**	**0.3**
T7 – T8	after third faricimab injection vs. 10-month FU	0.095	−0.2

bold: significant p-values

Measurements of SFCT at each time point are shown in Tables 4 and 5. Under prior treatment between initial diagnosis T1 and T4 (*p* = 0.305) and between T2 and T4 (*p* > 0.99) no significant SFCT reduction was achieved. During the faricimab loading phase (T4-T7) SFCT decreased significantly (*p* < 0.001). Six months after the loading phase at T8, SFCT did not improve significantly any further compared to T7 (*p* > 0.99). However, compared to T4, SFCT was still significantly reduced (*p* < 0.001, *r* = 0.4). Spearmann correlation showed no significant correlation between SFCT and VA, as well as SFCT and CST at all time point (T1-T8 *r* < 1; *p* > 0,05).

**Table 4:** Subfoveal choroidal thickness (SFCT) measurements during study period (T1-T8)

Time points	SFCT (µm) all eyes (*n* = 51)	SFCT (µm) ranibizumab subgroup (*n*= 14)	SFCT (µm) aflibercepts subgroup (*n*= 38)
T1	195.76 ± 85.76 183,00 (Q1:137,00; Q3:241,00)	189.09 ± 72.07 171,00 (Q1:125,00; Q3:248,00)	198.05 ± 91.14 188,00 (Q1:139,25; Q3:242,25)
T2	175.63 ± 78.49 161,00 (Q1:177,00; Q3:223,00)	174.15 ± 53.06 178,00 (Q1:135,00,; Q3:204,50)	176.13 ± 86.09 158,00 (Q1:110,75; Q3:226,25)
T3	173.29 ± 80.51 159,00 (Q1:114,00; Q3:220,00)	173.31 ± 61.13 159,00 (Q1:116,00; Q3:212,50)	173.29 ± 86.09 153,50 (Q1:105,75, Q3:223,00)
T4	172.33 ± 77.40 158,00 (Q1:116,00; Q3:219,00)	174.08 ± 58.02 175,,00 (Q1:126,50; Q3:218,50)	171.74 ± 83.68 155,50 (Q1:110,00; Q3:223,50)
T5	164.82 ± 81.61 146,00 (Q1:110,00; Q3:211,00)	163.15 ± 57.45 147,00 (Q1:118,50; Q3:211,00)	165.39 ± 89.04 146,00 (Q1:102,25; Q3:212,50)
T6	163.12 ± 87.18 143,00 (Q1:113,00; Q3:195,00)	155.54 ± 52.05 160,00 (Q1:116,00; Q3:194,00)	165.71 ± 96.77 140,50 (Q1:112,00; Q3:210,25)
T7	158.98 ± 83.10 141,00 (Q1:106,00; Q3:197,00)	150.69 ± 55.01 171,00 (Q1:101,50; Q3:186,00)	161.82 ± 91.20 139,50 (Q1:105,00; Q3:200,50)
T8	145.67 ± 70.85 127,00 (Q1:95,00; Q3:196,00)	140.38 ± 64.24 108,00 (Q1:108,00; Q3:174,00)	147.47 ± 73.69 128,00 (Q1:92,50; Q3:196,25)

top row: mean; bottom row: median (Q1 = 1^st^ Quartile; Q3 = 3^rd^ Quartile)

**Table 5:** Subfoveal choroidal thickness in pairwise comparison

Time points	Interpretation SFCT	Pairwise comparison, Asymptotic *p*- value	Effect power
T4 – T5	prior switch – after first faricimab injection	> 0.99	0.1
T4 – T7	prior switch – after third faricimab injection	0.001**	**0.3**
T4 – T8	prior switch – third faricimab injection vs. 10-month FU	<0.001**	**0.4**
T7 – T8	after third faricimab injection vs. 10-month FU	> 0.99	0.1

bold: significant *p*-values

VA *visual acuity*, FU *Follow up*

In a subgroup analysis, CST and SFCT were examined during the treatment period in dependence on whether the prior drug was ranibizumab or aflibercept right before the switch to faricimab. In total, 12 patients who had previously received ranibizumab (subgroup R), and 37 patients had previously received aflibercept (subgroup A) were included in the 6 month analysis. In subgroup R, CST did not decrease significantly between T4 and T8 (*p* = 0.948). However, in subgroup A, CST decreased significantly (*p* = 0.005). SFCT between T4 and T8 improved both for pretreatment subgroups R and A (subgroup R: *p* = 0.025; subgroup A: *p* < 0.001).

Baseline OCT showed PED in 49 eyes (96.1%), IRF in 18 eyes (35.3%) and SRF in all eyes.

**Fig. 3:**
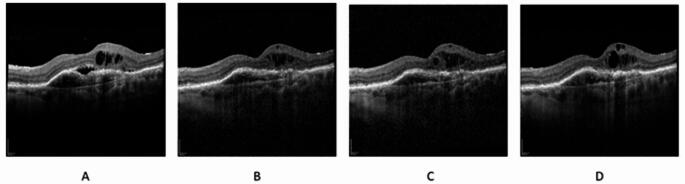
Optical coherence tomography example of weak treatment effect. A: Treatment naïve; B: T4 before switch to faricimab; C: T7 after third faricimab injection D: T8 six month after last faricimab injection. Patient was switched back to aflibercept and is currently under a four week treatment interval

**Fig. 4:**
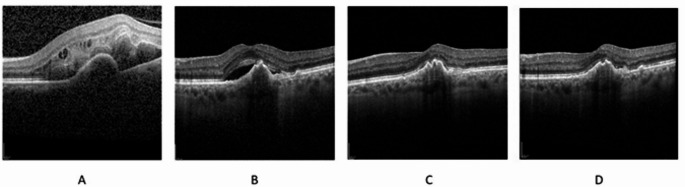
Optical coherence tomography example of strong treatment effect. A: Treatment naïve; B: T4 before switch to faricimab; C: T7 after third faricimab injection D: T8 six month after loading phase. Currently under a ten week treatment interval of faricimab

Pre-Switch OCT at T4 showed PED in 41 eyes (80.4%), IRF in 27 eyes (52.9%) and SRF in 39 eyes (76.5%). OCT at T8 showed PED in 28 eyes (54.9%), IRF in 14 eyes (27.5%) and SRF in 11 eyes (21.5%). In total, 33 out of 51 eyes (64.7%) had a completely dry macula after the third faricimab injection at T4. 27 out of 51 eyes (52.9%) had a completely dry macula at the end of follow up (T8). Figures 3 and 4 demonstrating examples of faricimab treatment effect in OCT.

### Visual acuity measurements

Visual acuity was maintained during faricimab treatment (0.30 (Q1:0.10 Q2:0.50) logMAR before switching vs. 0.30 (Q1:0.10 Q2:0.50) logMAR after switching; *p* = 0.07). Mean visual acuity measurements are shown in Table 6 and pairwise comparison between time point is demonstrated in Table 7.

**Table 6:** Visual acuity (VA) measurements during study period (T1-T8)

Time points	VA (LogMAR) all eyes	´VA (LogMAR) ranibizumab subgroup	VA (LogMAR) aflibercepts subgroup
T1	0.40 (Q1:0.20, Q3:0.78) *n*= 48/51	0.40 (Q1:0.23, Q2:0.90) *n*= 12/13	0.40 (Q1:0.20, Q3:0.76) *n*= 36/38
T2	0.30 (Q1:0.20, Q3:0.50) *n*= 51/51	0.30 (Q1:0.20, Q3:0.35) *n*= 13/13	0.30 (Q1:0.10, Q3:0.50) *n*= 38/38
T3	0.30 (Q1:0.20, Q3:0.50) *n*= 51/51	0.30 (Q1:0.10, Q3:0.30) *n*= 13/13	0.30 (Q1:0.20, Q3:0.50) *n*= 38/38
T4	0.30 (Q1:0.10 Q3:0.50) *n*= 51/51	0.30 (Q1:0.10 Q3:0.45) *n*= 13/13	0.30 (Q1:0.18, Q3:0.50) *n*= 38/38
T5	0.30 (Q1:0.10 Q3:0.40) *n*= 51/51	0.40 (Q1:0.20 Q3:0.40) *n*= 13/13	0.25 (Q1:0.10 Q3:0.43) *n*= 38/38
T6	0.50 (Q1:0.30 Q3:0.63) *n*= 51/51	0.40 (Q1:0.26, Q3:0.63) *n*= 13/13	0.50 (Q1:0.30, Q3:0.80) *n*= 38/38
T7	0.30 (Q1:0.20 Q3:0.50) *n*= 51/51	0.30 (Q1:0.20 Q3:0.45) *n*= 13/13	0.30 (Q1:0.16 Q3:0.50) *n*= 38/38
T8	0.30 (Q1:0.10 Q3:0.50) *n*= 51/51	0.30 (Q1:0.20 Q3:0.40) *n*= 13/13	0.20 (Q1:0.10 Q3:0.50) *n*= 38/38

(Q1 = 1^st^ Quartile; Q3 = 3^rd^ Quartile)

**Table 7:** Visual acuity (VA) measurements in pairwise comparison bold: significant p-values

Time points	Interpretation VA	Pairwise comparison, exact *p*- value	Effect power
T4 – T5	prior switch – after first faricimab injection	0.24	0.1
T4 – T7	prior switch – after third faricimab injection	0.57	0
T4 – T8	prior switch – third faricimab injection vs. 10-month FU	0.07	0.1
T7 – T8	after third faricimab injection vs. 10-month FU	0.22	0.1

VA *visual acuity*, FU *Follow up*

### Treatment intervals

The mean injection interval was significantly extended from 37.0 ± 9.5 days before the switch to 56.1 ± 30.4 days after the switch at the end of follow up (*p* = 0.004) at nine months, resulting in an extension of 51%. The 95% confidence interval for the mean difference in injection interval days was 26.88 ± 4.18, with a lower limit of 18.49 days and an upper limit of 35.27 days.

## Discussion

Our data indicate that faricimab can not only provide good anatomical efficacy in nAMD when switching treatment from ranibizumab or aflibercept, but also adds evidence that faricimab can provide greater durability than older anti-VEGF monotherapies. In this study, anatomical improvements on OCT obtained at three months after switching (the “loading phase”) were not only maintained at nine months, but treatment intervals using a regular treat & extend interval could also be prolonged by 51% - in spite of the difficult cohort of recalcitrant nAMD eyes with severe disease activity and a heavy treatment burden.

The new agent faricimab investigated in this study pursues a novel approach and combines two different means of treatment. There is preclinical evidence that affecting this second pathway may increase vascular stability and thereby reduce leakage [16], which was confirmed in large phase III clinical trials [9, 10]. These benefits might explain why durability was greater than the one observed with ranibizumab or aflibercept, two safe and effective older anti-VEGF agents which however do not modulate Ang2/Tie-2 signaling.

Registrational trials are an important source of information for approving new therapeutic agents. However, due to the strict study design and highly standardized therapeutic regimes, they might not adequately reflect real outcomes of actual patient treatment. Real-life studies have shown that 19–27% of eyes treated with ranibizumab or aflibercept required continuous four-week treatment intervals [17, 18]. This suggests a substantial group of patients could benefit from new therapies that offer better fluid reduction or longer treatment intervals. The phase 3 TENAYA and LUCERNE trials investigated the efficacy and durability of faricimab in treatment-naïve eyes with neovascular age-related macular degeneration (nAMD), demonstrating that faricimab was non-inferior to aflibercept with respect to both visual and anatomical outcomes, while allowing for extended treatment intervals of up to 16 weeks [11]. However, these pivotal studies were conducted exclusively in treatment-naïve populations, where more pronounced improvements in visual acuity (VA) and central subfield thickness (CST) are typically observed due to the absence of prior structural damage [19]. In contrast, our study cohort consisted of patients with recalcitrant nAMD who had previously undergone multiple anti-VEGF treatments and were switched to faricimab due to suboptimal response. These eyes often present with chronic disease features such as persistent fluid, macular fibrosis, or geographic atrophy, which may limit the potential for functional recovery and anatomical improvement.

Since its approval, faricimab has been investigated in several real-world studies for nAMD treatment, both in the short [19–21] and long term [13, 22]. Most short-term studies focus on treatment-naïve patients with follow-up periods of around three to four months, generally reporting promising anatomical improvements and stable or improved visual outcomes [21–26]. However, long-term real-world data remain limited due to the drug’s recent introduction.

The TRUCKEE study from the US demonstrated significant improvements in visual acuity and CST after six months of faricimab treatment. In contrast to our findings, continued improvements were observed after repeated injections [27]. Notably, only 25% of eyes in TRUCKEE received more than one injection, whereas all patients in our cohort underwent at least three. The criteria for additional injections were not specified. Furthermore, TRUCKEE reported a shorter average injection interval (~ 44 days) in pre-treated eyes compared to our study, although their six-month observation period likely contributed to this difference. Among studies with longer follow-up, Matsumoto et al. monitored 30 treatment-naïve eyes over 12 months and reported an average treatment interval of approximately 13 weeks [28]. This longer interval likely reflects the better anatomical response expected in treatment-naïve eyes [19]. Several studies have also assessed faricimab in pre-treated, resistant nAMD cases, showing short-term improvements in CST and VA along with extended treatment intervals [19, 25, 26]. In a Japanese cohort, Katayama et al. observed initial reductions in CST and SFCT following the first injection, but these changes were no longer statistically significant at six months [22]. Leung et al. reported CST improvements and extended intervals after eight months of treatment in pre-treated eyes [19], while Rush et al. found reductions in CST, gains in VA, and extended intervals beyond eight weeks in 31.5% of 54 pre-treated eyes after 12 months of treatment [29].

This analysis aimed to assess the long-term outcomes nine months after switching to faricimab from another anti-VEGF agent. We focused on two main aspects: morphological outcomes from OCT scans and functional outcomes measured by visual acuity. OCT, a standard diagnostic tool in AMD, allows for fast and accurate assessment of nAMD [6, 30]. CST, a key marker for quantifying CNV activity, provides a reliable reference point (6). We also analyzed SFCT, which is closely linked to nAMD pathophysiology, as choroidal vessel growth is influenced by VEGF mediated by the retinal pigment epithelium [16, 31, 32]. Previously, we demonstrated a significant decrease in CST and SFCT during the loading phase [33], both of which were maintained as the injection interval was extended from 35.80 ± 9.54 days to 56.17 ± 30.07 days after nine months. The literature presents mixed findings on SFCT changes during anti-VEGF treatment. VEGF inhibition may cause SFCT thinning through vasoconstriction and reduced choriocapillaris endothelial cell fenestrations, or it could be secondary to suppressed CNV activity [34]. Faricimab, an agent targeting both VEGF and Ang1/Tie2 pathways, may have a greater impact on nAMD pathophysiology than standard anti-VEGF treatments.

Published studies vary in their use of a loading phase before treatment extension, and limited long-term data makes comparisons challenging. Current literature supports an initial loading phase. While pre-approval studies recommend 4-week interval extensions after dry OCT, our patients received 2-week extensions per institutional protocols. Good outcomes with 2-week extensions are also reported [13, 19, 29]; only Matsumoto et al. used 4-week extensions in treatment-naïve eyes with favorable results [28]. Whether this is applicable to pretreated eyes remains unclear and requires long-term data. Our cohort had extensive pretreatment (mean 34.6 ± 25.1 anti-VEGF injections), yet showed limited prior response. Nonetheless, switching to faricimab improved CST and SFCT. SFCT, a marker of MNV perfusion, has been linked to treatment response in nAMD [35].

Our results align with previous findings, confirming significant CST reduction over a longer follow-up of nine months. Unlike most studies with up to six months’ follow-up, our data spans approximately nine months (188.40 ± 15.27 days) in a European cohort. Notably, CST improvements were most pronounced after the first injection, with later injections maintaining rather than enhancing the effect. Eyes previously treated with ranibizumab showed a weaker response than those switched from aflibercept, though this may reflect the smaller ranibizumab subgroup. Our current findings do not demonstrate superior visual acuity gains compared to previous treatments. Instead, the primary benefit observed was treatment durability, reflected in extended injection intervals and sustained anatomical outcomes. Although anatomical improvements were observed in patients treated with faricimab, no significant gains in visual acuity were noted. Chronicity of disease and the presence of irreversible retinal damage, such as photoreceptor atrophy, may limit the potential for visual recovery despite resolution of fluid. This discrepancy was previously already described in literature. These findings highlight the complex nature of nAMD and suggest that while anatomical improvement is a crucial marker, it may not always translate to functional recovery in patients with long-standing disease [36, 37]. The primary purpose of our study was not to evaluate potential side effects. Nevertheless, there were no severe ocular complications recorded during this study period (no cases of intraocular inflammation, retinal detachment, severe spikes in IOP, intraocular hemorrhages or tears of the retinal pigment epithelium). Retinal specialists currently have a variety of approved, highly effective nAMD therapies that have a good safety profile. New drugs always raise concerns about potential side effects, particularly intraocular inflammation, drug-induced retinal vasculitis and retinal artery occlusion. Any new therapy must therefore have a safety profile that is comparable with established standard treatments under real-life conditions. despite a good risk profile in the approval studies, real-word data describing possible side effects are therefore essential.

A limitation of this study is the lack of a control group continuing treatment with ranibizumab or aflibercept due to retrospective design of this study, which should be considered when interpreting the observed outcomes, as they may partly reflect the effects of ongoing anti-VEGF therapy rather than faricimab alone. The lack of a control group continuing treatment with ranibizumab or aflibercept due to the retrospective design prevents a direct comparison and limits the ability to attribute observed outcomes exclusively to faricimab. It remains uncertain whether improvements or stability in anatomical or functional outcomes are due to the new treatment or part of the natural variability under continued anti-VEGF therapy. The absence of randomization also introduces potential selection biases, as treatment decisions may have been influenced by clinical judgment, disease activity, or patient preference, which could confound the results. While our follow-up period of nine months exceeds that of several other real-world reports, longer observation periods will be essential to fully evaluate long-term safety outcomes such as macular atrophy, subretinal fibrosis, and the potential for delayed recurrence of disease activity. As a limitation, 91% of eyes completed the full faricimab series, with some not responding favorably and potentially benefiting from a return to prior treatment.

In summary, our data support the long-term efficacy of switching nAMD patients to faricimab over nine months, with major improvements seen primarily after the first injection. While no additional anatomical benefits were noted at nine months compared to three months, disease control was maintained with extended treatment intervals. However, these findings should be interpreted with caution given the retrospective, uncontrolled nature of the study and the relatively short follow-up. The absence of a parallel control group limits our ability to isolate the effects of faricimab from ongoing anti-VEGF treatment in general. Given the retrospective nature of our study, prospective randomized controlled trials are warranted to confirm the anatomical and functional benefits of switching to faricimab in recalcitrant nAMD. Furthermore, future research may focus on the identification of imaging biomarkers that could help predict which patients are most likely to respond favorably to faricimab, enabling a more personalized treatment approach.
